# Axonal Cleaved Caspase-3 Regulates Axon Targeting and Morphogenesis in the Developing Auditory Brainstem

**DOI:** 10.3389/fncir.2016.00084

**Published:** 2016-10-24

**Authors:** Sarah E. Rotschafer, Michelle R. Allen-Sharpley, Karina S. Cramer

**Affiliations:** Department of Neurobiology and Behavior, University of CaliforniaIrvine, CA, USA

**Keywords:** caspase, auditory, brainstem, development, hindbrain, magnocellularis, laminaris, astrocytes

## Abstract

Caspase-3 is a cysteine protease that is most commonly associated with cell death. Recent studies have shown additional roles in mediating cell differentiation, cell proliferation and development of cell morphology. We investigated the role of caspase-3 in the development of chick auditory brainstem nuclei during embryogenesis. Immunofluorescence from embryonic days E6–13 revealed that the temporal expression of cleaved caspase-3 follows the ascending anatomical pathway. The expression is first seen in the auditory portion of VIIIth nerve including central axonal regions projecting to nucleus magnocellularis (NM), then later in NM axons projecting to nucleus laminaris (NL), and subsequently in NL dendrites. To examine the function of cleaved caspase-3 in chick auditory brainstem development, we blocked caspase-3 cleavage in developing chick embryos with the caspase-3 inhibitor Z-DEVD-FMK from E6 to E9, then examined NM and NL morphology and NM axonal targeting on E10. NL lamination in treated embryos was disorganized and the neuropil around NL contained a significant number of glial cells normally excluded from this region. Additionally, NM axons projected into inappropriate portions of NL in Z-DEVD-FMK treated embyros. We found that the presence of misrouted axons was associated with more severe NL disorganization. The effects of axonal caspase-3 inhibition on both NL morphogenesis and NM axon targeting suggest that these developmental processes are coordinated, likely through communication between axons and their targets.

## Introduction

Caspase-3 is an evolutionarily conserved member of the cysteine-aspartic acid protease family that plays a well-established role in apoptosis (Kuida et al., 1996). Caspases are categorized as either initiator or effector caspases. Initiator caspases cleave effector caspases, which may then cleave other protein substrates in an apoptotic cascade. Caspase-3 exists in either an inactive pro-caspase form, or an active cleaved caspase form. Caspase-9, an initiator caspase, activates procaspase-3 by cleaving it into active caspase-3, which may then initiate cell death (Kuida et al., 1996; Salvesen and Dixit, 1997; Zou et al., 1997).

Caspase-3 induced apoptosis has been observed in neurons during programmed developmental cell death (Kuida et al., 1996; Jacobson, 1997; Pompeiano et al., 2000; Roth et al., 2000; Mayordomo et al., 2003), and is a necessary component for normal brain development (Kuida et al., 1996). Capase-3 deficient mice for instance, display hyperplasia throughout the brain and disorganized cell deployment (Kuida et al., 1996). Caspase-3 has also been shown to induce apoptosis in microglia and astrocytes of developing brains (Zhang et al., 2000; Dalmau et al., 2003).

More recently, non-apoptotic functions of caspase-3 have been discovered. Several studies have found caspase-3 to play an important role in cell differentiation in both neurons (Ishizaki et al., 1998; Fan et al., 2014) and glia (Oomman et al., 2005). Moreover, caspase-3 influences neuronal development by determining branch points in retinal axon arbors (Campbell and Okamoto, 2013) and pruning of dendrites and dendritic spines (Ertürk et al., 2014).

Caspase-3 is known to be necessary for the development of auditory function. Caspase-3 knockout mice (caspase-3^−/−^ mice) experience hyperplasia of supporting cells resulting in cochlear malformation, and generally immature cochlear morphology (Takahashi et al., 2001). There is an overall reduction in the number of inner and outer hair cells in caspase-3^−/−^ mice (Morishita et al., 2001), and cilia in remaining hair cells are often fused (Takahashi et al., 2001). Caspase-3 thus regulates the balance of cell types in the developing inner ear. Neuronal cells of the spiral ganglion degenerate in caspase-3^−/−^ mice, and hearing function is severely impaired (Morishita et al., 2001).

Caspase-3 has been shown to be a necessary component of peripheral auditory system development, but its role in the developing central auditory system is unknown. Here, we investigated caspase-3 expression and function during the maturation of the auditory brainstem. We used whole embryo chick cultures that permit access to the brainstem over the course of development. In the chick brainstem, the first stages of auditory processing are performed by nucleus magnocellularis (NM), a homolog of the anteroventral cochlear nucleus, and nucleus laminaris (NL), analogous to the medial superior olive (Smith and Rubel, 1979). Input from the peripheral auditory system is conveyed by central projections of cochlear ganglion cells through the auditory portion of the VIIIth cranial nerve. These projections form the first central auditory synapses, which terminate on NM neurons. Each NM neuron then sends out a bifurcating axon to NL. One branch crosses the midline and projects to the ventral portion of the contralateral NL. The other NM axon branch forms synapses on the dorsal side of the ipsilateral NL. This pathway integrates binaural auditory input as a means of performing sound localization (Carr and Konishi, 1990; Overholt et al., 1992). We found prominent expression of cleaved caspase-3 in the developing chick auditory brainstem with a temporal sequence that followed the ascending projection from the VIIIth nerve to NM and then to NL. To test the function of cleaved caspase-3 in development, we blocked caspase-3 cleavage in the developing chick brainstem over several days. Loss of caspase-3 activity resulted in disorganization of NL neurons, the appearance of small non-neuronal cells within the normally cell-free dendritic zone around NL, and aberrations in NL innervation. These observations suggest that axonal caspase-3 regulates multiple aspects of circuit formation at a time that precedes programmed cell death in the developing auditory brainstem.

## Materials and Methods

### Immunofluorescence

Fertilized brown Leghorn chicken eggs (*Gallus domesticus*) were obtained from AA Laboratories (Westminster, CA, USA). To initiate development, eggs were placed into a rotating incubator at 39°C. Tissue was collected from chick embryos at embryonic day (E)6, E7, E9, E10, E11 and E13. We used 3–5 embryos at each age for each antibody in our expression studies. Dissected brainstems were fixed with 4% paraformaldehyde (PFA) in phosphate buffered saline (PBS) for 45 min and placed in a 30% sucrose solution in PBS overnight at 4°C. Brainstems were cryosectioned at 14 μm and mounted on chrome-alum glass slides in a 1-in-4 series to permit multiple histological assays for each specimen. Slides were then dried on a 37°C slide warmer, and sections were surrounded with a hydrophobic Pap pen barrier. Slides were rinsed, in PBS, then antigen retrieval was performed using a 0.1% sodium dodecyl sulfate in PBS solution for 5 min. Slides were rinsed again in PBS, then blocking solution (4% normal goat serum and 0.1% Triton X in PBS) was applied for 1 h in a humid chamber. Primary antibodies (see below) were diluted in blocking solution, then incubated with sections for at least 10 h in a humid chamber. Slides were rinsed in PBS and secondary antibody applied. Slides were incubated for 1 h with AlexaFlour (Life Technologies #A11001, Carlsbad, CA, USA) secondary antibodies (goat anti-mouse Alexa 488; goat anti-mouse 647; goat anti-rabbit 488) diluted 1:500 in blocking solution. Slides were rinsed with PBS, cover-slipped with glycergel mounting medium (Dako #C0563, Carpinteria, CA, USA), then imaged at 20× on Zeiss Axioskop2 microscope using Axiovision software.

### Primary Antibodies

For cleaved caspase-3 immunofluorescence we used a rabbit polyclonal antibody (Cell Signaling Technology #9661, Danvers, MA, USA) and a rabbit monoclonal antibody (R&D Systems #AF835, Minneapolis, MN, USA) that were generated using distinct synthetic peptides corresponding to human cleaved caspase-3 diluted 1:200 in blocking solution. Both antibodies revealed similar patterns of cleaved caspase-3 expression. We also performed a negative control in which the primary antibody was omitted. The negative control did not show any immunofluorescence that corresponded with cleaved caspase-3 expression. To label procaspase-3 we used a rabbit polyclonal anti-procaspase-3 primary antibody (Abcam ab90437, Cambridge, MA, USA) diluted 1:500 in blocking solution. We used a mouse monoclonal neurofilament (NF) primary antibody (EDM Millipore MAB5266, Darmstadt, Germany) diluted 1:500 in blocking solution in conjunction with cleaved caspase-3 immunolabeling. In our caspase-3 inhibition studies (see below), we found that the inhibitor reduced expression of cleaved caspase-3 but not procaspase-3, providing additional support for the specificity of these antibodies. We performed immunofluorescence for the astrocyte marker aldehyde dehydrogenase 1 family member L1 (ALDH1L1) using a mouse monoclonal antibody (Abcam ab56777, Cambridge, MA, USA) diluted 1:500 in blocking solution. In embryos used in axon tracing studies (see below), we performed immunofluorescence for microtubule-associated protein 2 (MAP2), which is expressed throughout NL dendrites and can thus be used to outline the extent of NL neuropil (Wang and Lenardo, 2000; Person et al., 2004; Wang and Rubel, 2008; Tabor et al., 2011). The blocking solution was 10% bovine serum albumin, 10% heat inactivated normal goat serum, 0.05% Triton X in PBS. Primary mouse anti-MAP2 monoclonal antibody (Millipore MAB3418, Temecula, CA, USA) was diluted to 1:200 in blocking solution.

### Nissl Staining

To visualize cell bodies, we performed fluorescent Nissl staining using the BrainStain Imaging Kit (Life Technologies #B34650, Carlsbad, CA, USA). Mounted sections prepared as previously described were outlined with a hydrophobic pap pen barrier, then washed in PBS and 0.2% Triton X solution. Slides were incubated at room temperature for 20 min with NeuroTrace 530/615 red fluorescent Nissl stain (Life Technologies #B34650, Carlsbad, CA, USA), diluted 1:300 in PBS. Immunofluorescence was then performed as previously described. For brightfield Nissl staining, mounted cryosections were stained with 1% thionin, dehydrated through a graded series of alcohol and then xylene, and coverslipped with DPX mounting medium.

### Whole Embryo Cultures

Fertilized brown Leghorn chicken eggs (*Gallus domesticus*) were obtained from AA Laboratories (Westminster, CA, USA). To initiate development, eggs were placed into a rotating incubator at 39°C. At E3, the eggs were cracked and all egg contents, including the chicken embryo were very carefully transferred into a small dish with a cover to prevent dehydration. Whole embryo cultures were then maintained in a dark non-rotating incubator at 39°C for the duration of the experiment.

### Z-DEVD-FMK Injections

At 3 days *in vitro* (DIV), embryos were staged at embryonic day 6 (E6) and continued to proceed through development along a time course similar to that seen *in vivo* (Allen-Sharpley and Cramer, 2012); we refer to these embryos as E6. Beginning at this age, we made daily injections of sham, control, or Z-DEVD-FMK solution into the IVth ventricle of cultured embryos. Z-DEVD-FMK (BD Pharmingen #550378, San Jose, CA, USA) is a tetrapeptide that selectively inhibits caspase-3 cleavage. Z-DEVD-FMK was reconstituted in dimethyl sulfoxide (DMSO) to make a 10 mM stock solution. The stock solution was then diluted using sterilized PBS containing NaCl and Na_3_PO_4_ to a final concentration of 50 μM. Because Z-DEVD-FMK was reconstituted in DMSO, a DMSO control solution was made by adding 10 μL DMSO to 200 μL sterilized PBS. Sterilized PBS was used as a sham control solution. A small amount of methylene blue was added to each solution to confirm placement of solution in the correct location. Injections were delivered through a 1.2 mm-diameter pulled glass pipette attached to a Picospritzer. Incubation was repeated over several days and injection volume was increased each day to fill the increasing volume of the brainstem and IVth ventricle. At E6, an average of 18.9 μL was injected into each embryo; at E7 an average of 22.9 μL was injected; at E8 an average of 53.3 μL was injected and at E9 an average of 67.6 μL was injected. At E10, brainstems were dissected and processed for imaging and analysis.

To test the efficacy of Z-DEVD-FMK in blocking caspase-3 cleavage, tissue from five control animals and six Z-DEVD-FMK injected embryos was immunolabeled with cleaved caspase-3 antibody. We quantified the reduction in cleaved caspase-3 expression by finding the optical density of immunolabel in NM using the measure function in ImageJ (NIH). We normalize it to the optical density of unlabeled background in a region adjacent and slightly dorsolateral to NM, which has a similar proximity to the injected IVth ventricle.

### Analysis of NL Morphology

Nissl-stained sections containing NL were imaged in brightfield at 20× on a Zeiss Axioskop2 microscope using Axiovision software. At least two brainstem sections were analyzed for each embryo. We used 13 shams, 17 controls and 23 Z-DEVD-FMK injected embryos for this analysis.

To describe how well neuronal cells within NL formed a single-cell thick lamina, *X − Y* coordinates (medial-lateral distance in μm by dorsal-ventral distance in μm) were assigned to neurons within NL using *X − Y* data from the cell counter function of ImageJ. NL neurons were identified as intensely labeled large cells, approximately 20 μm in diameter (Smith and Rubel, 1979). A regression line was fit to those coordinates and the *R^2^* coefficient found for each regression line. Using the regression line as a centralized point of reference within each NL, we imposed a parallel line 40 μm dorsal to the regression line and a parallel line 40 μm ventral to the regression line. Because Z-DEVD-FMK treatment could dramatically disrupt lamination of NL, the spaces between the regression line and the imposed lines acted to consistently mark the dorsal and ventral cell-free dendritic zone in all nuclei analyzed. The number of smaller cells intruding into the dorsal cell-free dendritic zone (the space between the regression line and the dorsal imposed line) and into the ventral cell-free dendritic zone (the space between the regression line and the ventral imposed line) were counted and compared.

### Axon Tracing

Brainstems were dissected from sham, control and Z-DEVD-FMK injected chick brainstems at E10 and maintained in an oxygenated artificial cerebrospinal fluid (ACSF) solution containing 10% ACSF stock solution (125.0 mM NaCl, 2.5 mM KCl, 25.0 mM NaHCO_3_, 1.25 mM KH_2_PO_4_, 10.0 mM glucose), 0.12% 1 M MgSO_4_ and 0.24% 1 M CaCl_2_ for up to 30 min. Brainstems were then injected with a small amount of rhodamine dextran amine (RDA) in PBS with 1% Triton X-100 at the midline, where branched of NM axons cross to the contralateral NL. Brainstems were then returned to the oxygenated ACSF solution for another 30 min to allow anterograde transport of RDA to axon terminations in the ventral NL. Brainstems were then post-fixed with 4% PFA in PBS for 45 min and placed in a 30% sucrose solution in PBS overnight in a 4°C refrigerator. Brainstems were cryosectioned at 14 μm and mounted.

### Analysis of Axon Tracing

We obtained 20× images of RDA tracing co-labeled with MAP2 immunofluorescence in NL using a Zeiss Axioskop2 microscope using Axiovision software. MAP2 staining highlighted NM and NL cell bodies, while RDA injections into midline crossing axons revealed NM axon terminations in contralateral NL. Normal targeting of these crossed projections is limited to the ventral dendrites and cell bodies of NL. We thus categorized each specimen as having “axon targeting errors,” in which axons and axon fascicles were seen coursing into dorsal NL dendrites or having “no axon targeting errors,” in which axon projections labeled from the midline were limited to the ventral region of NL. This categorization was made for axon tracts with fascicles of NM axons growing together toward NL. A minimum of three sections was scored for each animal. Sham and control were pooled in this analysis. Eight sham and control animals, and eight Z-DEVD-FMK animals were used in this analysis.

### TUNEL Labeling

We performed terminal deoxynucleotidyl transferase dUTP nick end labeling (TUNEL), which reveals fragmented DNA generated during apoptosis, to determine whether cleaved caspase-3 expression coincides with developmental programmed cell death. We used brainstem tissue from E8, E10 and E13 chick embryos. Three brainstems were collected for each embryonic age. Brainstems were fixed in 4% PFA then placed in a 30% sucrose solution overnight. Cryosectioned 8 μm-thick coronal sections were mounted on chrome-alum glass slides and dried on a 37°C slide warmer.

We used the Promega DeadEnd Fluorometric TUNEL System (Promega #G3250, Madison, WI, USA), with minor modifications to the protocol provided. Briefly, slides were rinsed in PBS, post-fixed 4% PFA for 15 min, then rinsed twice more. To permeabilize sections, E10 and E13 slides were placed in a 1% SDS/0.2% Triton-X 100 in PBS solution for 5 min and rinsed twice in PBS. E8, E10 and E13 slides were then incubated with 20 μg/ml proteinase K (Promega #V302A, Madison, WI, USA) in PBS for 20 min. Slides were rinsed, then placed in 4% PFA for 5 min. Slides used for the positive control group were incubated in a humid chamber at room temperature with DNase I buffer containing 40 mM Tris-HCl, 10 mM NaCl_2_, 6 mM MgCl_2_ and 10 mM CaCl_2_ for 5 min. DNaseI buffer was removed from slides and 100 μl of 10 μl/mL DNase I (Promega #M6101, Madison, WI, USA) in DNase I buffer was applied for 10 min. Slides were washed three times in deionized water and all additional processing of control slides was done in a dedicated Coplin jar in order to prevent cross-contamination with the test and negative control groups.

Slides were incubated for 10 min in a humid chamber with 100 μl of equilibration buffer (Promega #G327C, Madison, WI, USA). Positive control and test slides were then incubated under a plastic coverslip in a solution containing 45 μl equilibration buffer, 5 μl fluorescein labeled nucleotide mix (Promega #G328A, Madison, WI, USA), and 1 μl terminal deoxynucleotidyl transferase (rTdT) enzyme (Promega #M828B, Madison, WI, USA) for 1 h. For negative control slides, we used the same conditions but we omitted the rTdT enzyme. All slides were rinsed for 15 min in 2× saline sodium citrate buffer (Promega #G329A, Madison, WI, USA) to terminate the reaction then washed in PBS. Slides were mounted using ProLong Gold antifade reagent with 4′,6-diamidino-2-phenylindole (DAPI) (LifeTechnologies #P36931, Eugene, OR, USA) and imaged at 20× on a Zeiss Axioskop2 microscope using Axiovision acquisition software.

Images were analyzed for the percentage of cell nuclei positive for TUNEL labeling using the cell counter tool in ImageJ. The auditory anlage was identified in E8 images, and the NM and NL identified in E10 and E13 images. Color channels were split, and the number of DAPI-labeled nuclei and TUNEL-positive nuclei were counted. The percentage of nuclei that were TUNEL-positive was then calculated. Three brainstems were examined for each age group.

## Results

### Cleaved Caspase-3 Expression Ascends the Auditory Brainstem Pathways

**Figure 1:**
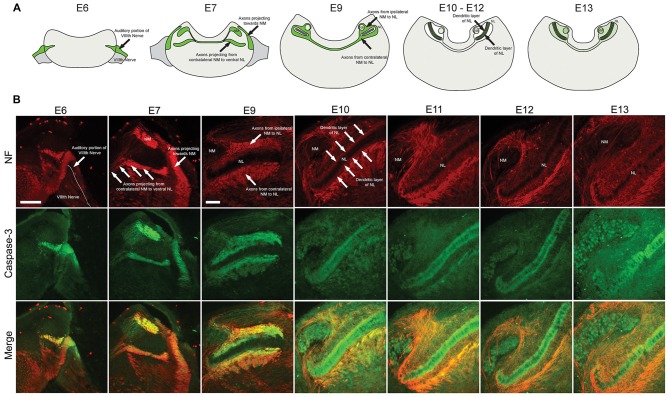
**Spatial and temporal expression of cleaved caspase-3 in the developing chick auditory brainstem. (A)** Summary of cleaved caspase-3 expression (shown in green) from E6 to E13. **(B)** Expression of cleaved caspase-3 in the brainstem from E6 to E13. Neurofilament (NF) staining (red) shows the developmental progression of axonal tracts, with cleaved caspase-3 expression (green) closely following axonal tract development (merged). At E6 labeling is seen in the peripheral and central projections of the auditory VIIIth nerve fibers. At E7 central projections of auditory nerve fibers and their synaptic target, nucleus magnocellularis (NM), also shows labeled axons. At E9–10 NM axons are labeled. At E11–12 these NM fibers are no longer labeled, but expression is seen in dendrites of their synaptic target, nucleus laminaris (NL). Scale bar for E6 = 100 μm, applies to E6–7. Scale bar for E9 = 100 μm, applies to E9–13.

Coronal brainstem sections from E6, E7, E9, E10, E11 and E13 chick embryos were immunolabeled for cleaved caspase-3 together with NF, a structural protein found in axons (Figure 1). Similar patterns were observed using both cleaved caspase-3 antibodies, summarized in Figure 1A. Central projections from the auditory portion of the VIIIth nerve have been previously defined within NF immunolabeled regions (Allen-Sharpley et al., 2013). At E6, prior to the formation of NM and NL from the auditory anlage in the brainstem, cleaved caspase-3 was expressed along the dorsolateral portion of the VIIIth nerve, consistent with the auditory and not the vestibular portion (Figure 1B). At E7, cleaved caspase-3 was expressed along axons projecting to the dorsolateral portion of the brainstem where auditory anlage had begun to form (Figure 1B). Additionally, cleaved caspase-3 was expressed in axons projecting from the developing NM to the developing NL on both sides of the brainstem.

By E9, NM and NL have separated from one another. NM axons projecting bilaterally to the dorsal portion of ipsilateral NL and to the ventral portion of contralateral NL were identifiable. Cleaved caspase-3 was expressed in these NM-NL axons (Figure 1B). Although refinement of NM and NL continues into later developmental stages, by E10 NL had achieved its characteristic morphology with a single-cell thick lamina of principle neurons surrounded by a cell-free dendritic zone. A layer of glial cells then surrounds NL neurons along both its dorsal and ventral portions.

At E10, caspase-3 expression within axons innervating NL was noticeably reduced (Figure 1A). Rather, cleaved caspase-3 expression was most apparent within the dendrites of NL neurons. Cleaved caspase-3 expression continued to be most prominent in NL dendrites at E11 and E13 (Figure 1B). After these ages caspase-3 expression in other portions of the brainstem becomes more prominent, consistent with previous reports of cell death in later embryonic development (Rubel et al., 1976). For the observed embryonic ages, cleaved caspase-3 expression very precisely follows the spatial and temporal development of the auditory brainstem.

### Axonal Cleaved Caspase-3 Expression Precedes Programmed Cell Death in NM and NL

**Figure 2:**
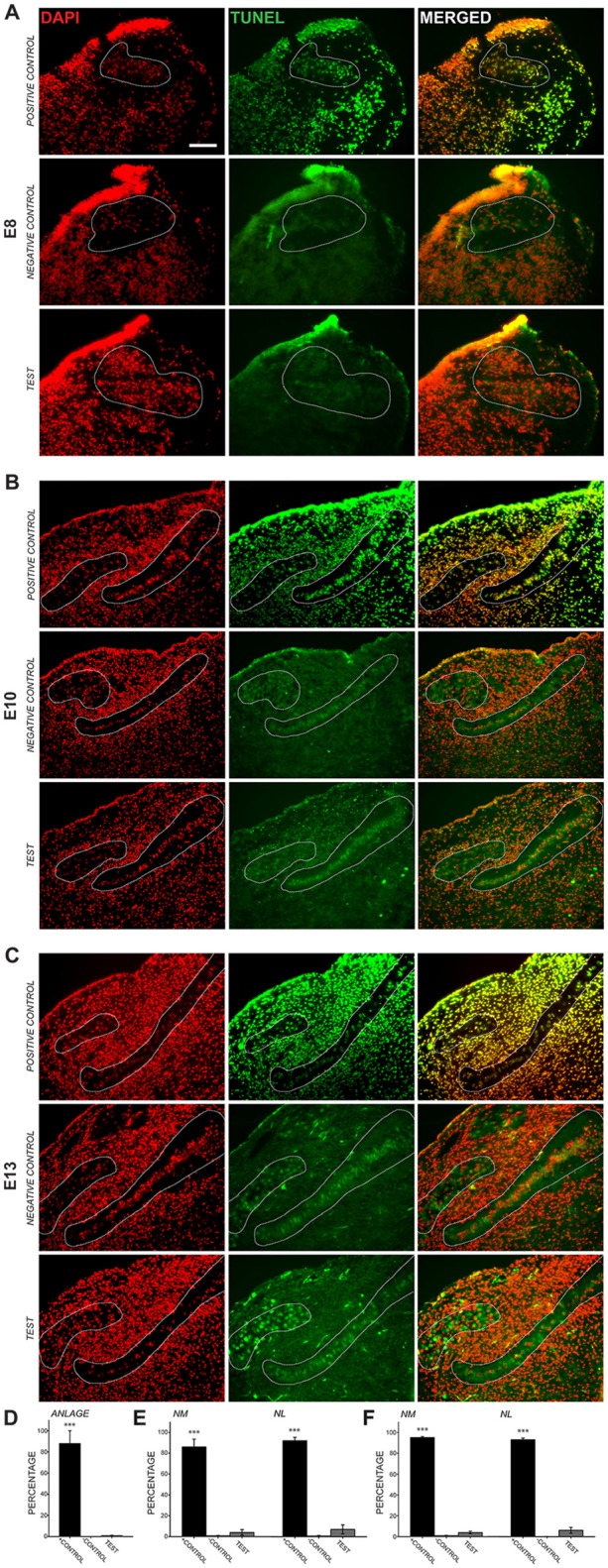
**Transferase dUTP nick end labeling (TUNEL) assessment of development cell death.** 4′,6-diamidino-2-phenylindole (DAPI) labels all cell nuclei, while TUNEL labels nicked DNA characteristically seen during apoptosis. **(A)** At E8, NM and NL have not completely separated from one another and are found in the auditory anlage (dashed lines). Positive control sections show nearly perfect alignment of DAPI-labeled cell nuclei and nuclei positive for TUNEL labeling (top row). Negative control sections did not show TUNEL-positive nuclei aligned with DAPI-labeled nuclei (middle row). Labeling in test sections (bottom row) revealed few, if any TUNEL-positive nuclei in the auditory anlage. Scale bar = 100 μm, applies to **(A–C)**. **(B)** TUNEL labeling in E10 sections showed few positive nuclei in NM or NL. **(C)** Test sections from E13 animals showed sparse nuclear TUNEL-labeling in NM and NL. Cytoplasmic TUNEL labeling was seen at this age. **(D–F)** Histograms showing the percent of DAPI-labeled nuclei that co-localized with nuclear TUNEL labeling in the auditory nuclei at each age. Percentages in the positive control were significantly greater (***p < 0.001; one-way ANOVA) than in negative control or test sections, which did not differ from each other.

Previous studies using cell counts have shown that developmental declines in NM and NL cell number occur after E11 (Rubel et al., 1976), suggesting that the cleaved caspase-3 we observed in auditory axons is not primarily associated with apoptosis. To ascertain whether cleaved caspase-3 expression in axons precedes the period of programmed cell death, we performed TUNEL labeling on E8, E10 and E13 chick brainstems (Figure 2). Consistent with these previous findings, few, if any TUNEL-positive cells were apparent in auditory brainstem nuclei in the sections we tested at E8, similar to negative controls and in contrast to positive controls (Figure 2A). Similarly, at E10, few TUNEL-positive cells were found in the test sections (Figure 2B). At E13 (Figure 2C), TUNEL labeling was seen in NM cells, where it appeared primarily in a cytoplasmic distribution at greater levels than in negative controls. These labeled cells were also seen in NL, but more sparsely; few cells showed nuclear labeling in either NM or NL.

We quantified the percent of cell nuclei in NM and NL that showed positive TUNEL labeling (Figures 2D–F). At E8 0.81 ± 0.36% of DAPI-labeled nuclei in the auditory anlage were TUNEL labeled. No cells were TUNEL-positive in the negative controls and 87.9 ± 12.1 (S.E.M.)% of the cells were TUNEL-positive in the positive control. A one-way ANOVA comparing TUNEL-positive cells in the positive control, negative control and test group showed no difference between the negative control and test groups, although both were significantly different than the positive control group (*F*_(2,4)_ = 74.730, *p* < 0.001). At E10 (Figure 2E), few TUNEL-positive cells were found in the test sections (NM: 3.99 ± 2.71%; NL: 7.00 ± 4.38%; *n* = 3). E10 negative controls showed no significant differences from our test sections, but both were significantly different than the control group (NM: 0.81 ± 0.28%; one-way ANOVA *F*_(2,5)_ = 167.515, *p* < 0.001; NL: 0.75 ± 0.42%; one-way ANOVA *F*_(2,5)_ = 213.523, *p* < 0.001; *n* = 3). Positive controls showed extensive label, with 86.1 ± 7.48% of NM cells and 92.1 ± 3.22% of NL cells TUNEL-positive. Thus, at E8–E10, when cleaved caspase-3 is seen in NM axons, there is no evidence of programmed cell death in NM and NL. At E13, while cytoplasmic labeling was seen abundantly in the test sections, only 3.9 ± 1.2% of NM cell nuclei and 6.1 ± 2.8% of NL cell nuclei were positive for TUNEL labeling (Figure 2F). These values did not differ from E13 negative controls, which showed 0.94 ± 0.38% of NM cells and 0.1 ± 0.1% of NL cells to be positive for TUNEL labeling, while more than 90% were TUNEL labeled in the positive control. The positive control group was significantly different than the negative control and test group (NM: one-way ANOVA *F*_(2,4)_ = 241.633, *p* < 0.001; NL: one-way ANOVA *F*_(2,4)_ = 441.916, *p* < 0.001; *n* = 3 in each group).

### Blocking Caspase-3 Cleavage Disrupts the Morphology of Nucleus Laminaris

We next tested the function of caspase-3 in the development of the NM-NL pathway using application of a blocking peptide, Z-DEVD-FMK. We specifically focused on ages during which NL and NM separate, NL forms a clear lamina and NM axons grow to appropriate regions of NL. We thus used daily injection of Z-DEVD-FMK into the fourth ventricle in whole embryo cultures from E6 and E10, when caspase-3 was expressed and TUNEL labeling was not apparent.

**Figure 3:**
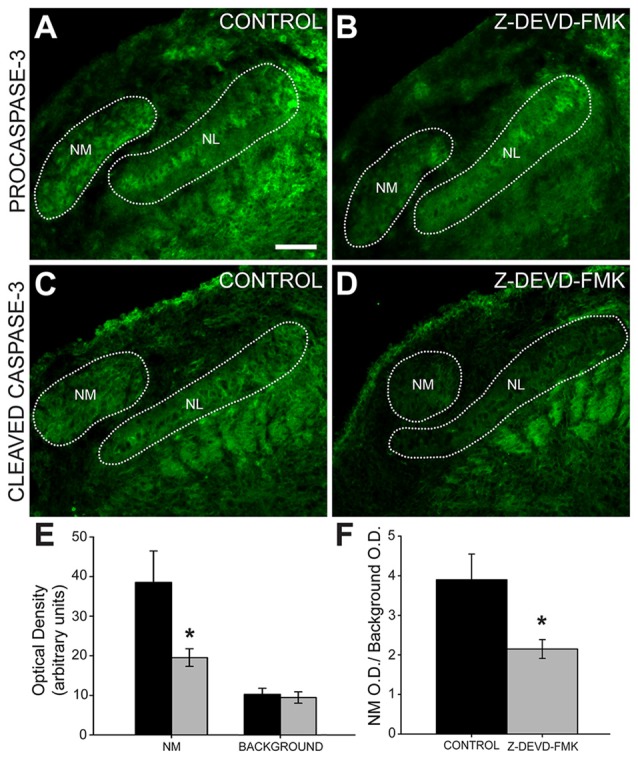
**Cleaved caspase-3 expression is reduced in Z-DEVD-FMK injected animals. (A)** NM cell bodies, NL cell bodies, NL dendrites and cells in the glial margin express procaspase-3 in control embryos and **(B)** Z-DEVD-FMK injected embryos. **(C)** In tissue from control animals, cleaved caspase-3 is expressed in NM and in NL dendrites. **(D)** In tissue from embryos that received injections of Z-DEVD-FMK into the IVth ventricle, cleaved caspase-3 expression was reduced. **(E)** While background optical density values did not differ, values in NM were significantly lower in treated vs. control embryos (*p < 0.5, *t*-test). **(F)** Quantification of cleaved caspase-3 expression showed that optical density measured in NM was significantly reduced in Z-DEVD-FMK injected embryos relative to background levels in an unlabeled area dorsolateral to NM (*p < 0.5, *t*-test). Scale bar in **(A)** = 100 μm, applies to **(A–D)**.

To ascertain whether injection of Z-DEVD-FMK inhibited caspase-3 in our preparation, we tested for the presence of pro- and cleaved caspase-3 using immunofluorescence (Figure 3). In sections taken from control animals, we saw procaspase-3 expressed in NL cell bodies, NL dendrites, the glial margin and in NM cell bodies (Figure 3A). Procaspase-3 expression in Z-DEVD-FMK injected tissue was similar to controls (Figure 3B). In control animals, cleaved caspase-3 was expressed in the NM and NL dendrites (Figure 3C). In sections taken from Z-DEVD-FMK injected embryos, cleaved caspase-3 expression was greatly reduced in both NM and NL (Figure 3D), which are both found dorsally near the injection site in the IVth ventricle. There was no significant difference in background optical density (*t*-test, *p* > 0.706; *n* = 5 treated and 6 controls). Optical density in NM was significantly reduced in the Z-DEVD-FMK injected group compared to controls (*t*-test, *p* < 0.05; Figure 3E).

We quantified the optical density of cleaved caspase-3 immunofluorescence in NM compared to background (NM optical density/background optical density). Cleaved caspase-3 expression is significantly reduced in Z-DEVD-FMK injected embryos (ratio = 2.15 ± 0.24) compared to controls (ratio = 3.9 ± 0.65, *t*-test, *p* < 0.05; Figure 3F). Based on this reduction in cleaved caspase-3 expression following Z-DEVD-FMK injections, we conclude that Z-DEVD-FMK successfully penetrated the dorsal brainstem into the auditory brainstem nuclei and significantly reduced expression of cleaved caspase-3.

**Figure 4:**
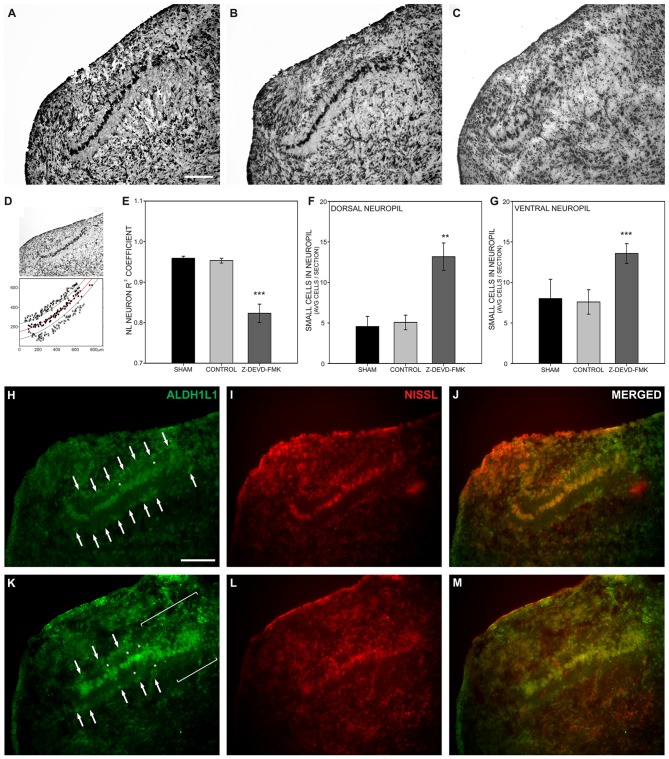
**Loss of cleaved caspase-3 impairs NL lamination. (A)** NL lamination appeared normal in Nissl stained sections from sham injected embryos and **(B)** control embryos. **(C)** In Nissl stained sections from Z-DEVD-FMK injected embryos, disruption of the NL lamina and surrounding cell-free neuropil was evident. **(D)** The extent of NL neuronal disarray was described using *R^2^* coefficients and by quantifying the extent of small cells permitted into the normally cell-free dendritic zone of NL. *R^2^* coefficients were found by assigning *X − Y* coordinates to NL neurons (black circles) and drawing a regression line through those points (red line). Lines parallel to the regression line were drawn 40 μm dorsal and ventral to the regression line to delineate the cell-free neuropil ended (black lines). The number of small cells (*x*’s) that normally remain outside the cell-free neuropil were quantified in control and treated embryos. **(E)** The Z-DEVD-FMK injected group had significantly lower *R^2^* coefficient values (***p < 0.001, one-way ANOVA), reflecting more neuronal disorganization. **(F,G)** Significantly more cells intruded into the dorsal **(F)** and ventral NL **(G)** neuropil in the Z-DEVD-FMK injected group than in controls (**p < 0.01; ***p < 0.001). The small cells surrounding NL in control animals (white arrows, **H–J**) express the astrocyte marker aldehyde dehydrogenase 1 family member L1 (ALDH1L1). In Z-DEVD-FMK injected embryos, ALDH1L1 was also expressed in the small cells surrounding NL (white arrows, **K–M**), in cells intruding into the NL neuropil (white asterisks, **K–M**), and in areas of NL where lamination was unclear (white brackets, **K–M**). Scale bar = 100 μm, applies to **(A–C,H–M)**.

To examine the effect of caspase-3 inhibition on NL development, Nissl stained brainstem sections containing NL were imaged (Figures 4A–C). Using those images of NL, *X − Y* coordinates (medial-lateral distance in μm by dorsal-ventral distance in μm) were assigned to neurons within NL (black circles, Figure 4D). A regression line was fit to those coordinates and the *R^2^* coefficient found for each regression line (red line, Figure 4D). In typically developing NL, primary neuronal cells form a single-cell thick tightly grouped sheet, and are described with an *R^2^* coefficient near 1, indicative of a strong linear fit. In the sham and control groups of embryos the mean *R^2^* coefficient was 0.950 ± 0.0132 (s.e.m.) and 0.951 ± 0.0068, respectively. The *R^2^* coefficient in the Z-DEVD-FMK injected group was 0.806 ± 0.0256. A one-way ANOVA showed a significant difference between groups *F*_(2,39)_ = 17.206, *p* < 0.001. Pairwise-comparison revealed no significant difference between sham and control group *R^2^* coefficients (Figure 4E). Both groups were significantly different than the *R^2^* coefficients of the Z-DEVD-FMK injected group.

Given the disruption of the neuronal lamina, we also investigated the effect of blocking caspase-3 cleavage on non-neuronal lamination within NL. At E10 the single-cell thick layer of neuronal cells within NL is normally surrounded by a cell body-free dendritic zone, which in turn is surrounded by a margin of glial cells, characterized by their small size and dense packing. This glial margin appeared disrupted in Nissl stained sections of treated embryos. To quantify this disruption of the glial boundary, we counted the number of small cells appearing ectopically in the cell-free dendritic zone. While neurons, dendritic zone and glial margin were clearly identifiable in most sections, the demarcation between dendritic zone and glial margin was degraded in other sections. We used the regression lines drawn to describe the neuronal distribution within NL in each nucleus analyzed. We then imposed a dorsal boundary for the cell-free dendritic zone 40 μm from the regression line and a ventral boundary 40 μm from the regression line (black lines, Figure 4D). We counted small cells found within the dorsal and ventral cell free dendritic zones and compared this number between treatment groups (Figure 4D). A one-way ANOVA revealed significant differences in the number of cells permitted into the dorsal zone (*F*_(2,37)_ = 5.249, *p* < 0.01) and into the ventral zone (*F*_(2,37)_ = 10.789, *p* < 0.001). Pairwise comparisons did not show any significant differences between the sham or control groups in this number. However, the number of non-neuronal cells intruding into both the dorsal and ventral cell-free dendritic zone was significantly different in the Z-DEVD-FMK injected group as compared to the sham and control groups (Figures 4F,G). When caspase-3 cleavage was blocked, NL neurons failed to form a single-cell thick lamina, and small cells distinct from NL principal neurons were found in the nucleus. These data suggest that cleaved caspase-3 is necessary for NL lamination and formation of the cell-free surrounding area.

In an effort to identify the small cells intruding into the cell-free dendritic space surrounding NL neurons, we performed immunofluorescence on sections from control (Figures 4H–J; *n* = 4) and Z-DEVD-FMK injected embryos (Figures 4K–M; *n* = 5) for the astrocyte marker ALDH1L1 (Figures 4H,K). Fluorescent Nissl staining allowed us to image cell bodies, NL morphology, and to identify small cells intruding into the cell-free dendritic zone of NL (Figures 4I,L). ALDH1L1 staining revealed the majority of cells surrounding NL to be astrocytes (Figures 4H,K). Moreover, treated specimens showed a reduced glial margin and the cells intruding into the NL cell-free dendritic zone expressed ALDH1L1 (Figures 4H,K), particularly in portions of NL where lamination was degraded (Figure 4K).

### Caspase-3 Blockage Drives Errors in Axonal Targeting and is Associated with Disrupted NL Morphology

**Figure 5:**
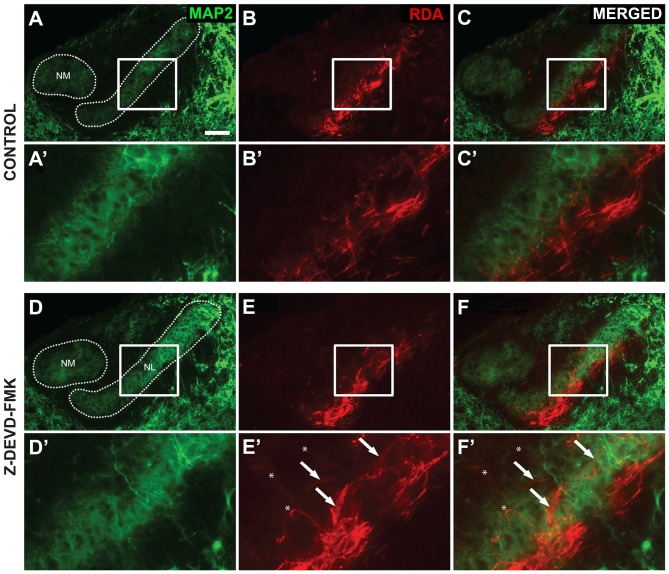
**Axon tract targeting is impaired in Z-DEVD-FMK injected embryos. (A)** Microtubule-associated protein 2 (MAP2) immunolabel shows the extent of NM and NL in a control embryo. **(A′)** Higher power shows inset. **(B,B′)** The same section showing rhodamine dextran amine (RDA) labeling from the midline in revealing axon terminations in NL. **(C,C′)** Merged images demonstrated that these terminations are appropriately limited to the ventral portion of NL. **(D,D′)** MAP2 immunolabel in a Z-DEVD-FMK injected embryo. **(E,E′)** RDA labeling from midline in the section shown in **(D)**. Axon arbors deviate from the normally confined ventral region. Axon tracts (white arrows) and individually resolvable axons (asterisks) were observed. **(F,F′)** Merged image shows the extent of mistargeting in the NL region. Scale bar in **(A)** = 100 μm, applies to **(A–F)**.

**Figure 6:**
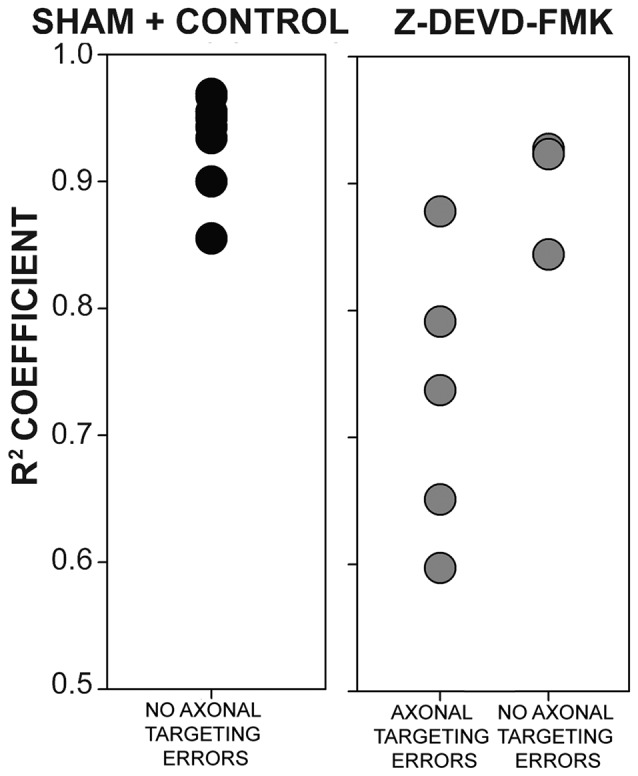
**The presence of axonal tract intrusions into NL was associated with greater disorganization of NL lamina.** No instances of axonal tract intrusion were seen in the sham + control group, and *R^2^* coefficient values were correspondingly high. High *R^2^* coefficient values describe NL with highly organized neuronal deployment. Several instances of axon targeting errors were seen in the Z-DEVD-FMK injected group, although not in all specimens. In instances of targeting errors, *R^2^* coefficient values were significantly lower than in instances of no axonal targeting errors in sham + control or Z-DEVD-FMK injected groups.

As blocking caspase-3 activity in the auditory axons of the developing chick brainstem results in poor lamination and gaps between NL cells, we sought to determine whether the disruptions in NL lamination may be associated with errors in axonal targeting (Parks and Rubel, 1975; Cramer et al., 2000; Huffman and Cramer, 2007). By E10, the dendrites along the ventral side of NL neurons have been innervated by the axons of the contralateral NM. We injected RDA into the midline crossing axons of E10 sham, control and Z-DEVD-FMK injected brainstems, and neurons within NL were imaged using MAP2 immunofluorescence. NL containing sections were scored according to whether or not axon tracts had permeated into or through the neuronal lamina of NL (Figure 5). Sections taken from sham or control embryos generally demonstrated a single cell-thick neuronal lamina (Figures 5A,A′), with axons terminating in the ventral NL dendrites or on the ventral portions of NL cell bodies (Figures 5B,B′,C,C′). Z-DEVD-FMK injected sections generally had less well defined neuronal NL lamina (Figures 5D,D′), as well as axons (asterisks, Figures 5E,E′,F,F′) and fascicles (arrows, Figures 5E,E′,F,F′) that were misrouted through NL. Because there were no significant differences between sham and control NL in *R^2^* values, ventral non-neuronal cell intrusion, or dorsal non-neuronal cell intrusion, we pooled sham and control data for this analysis. Eight sham + control animals and eight Z-DEVD-FMK animals were obtained. Of the eight embryos examined in the sham + control, there were no examples of axons interposed throughout the neuronal lamina of NL. However, of the eight Z-DEVD-FMK embryos tested, five exhibited growth of axonal fascicles beyond the neuronal lamina of NL, while three did not. The aberrant axons appeared to grow through the gaps in the NL lamina. To determine whether these errors in axonal targeting correlated with impaired NL morphology, we performed a one-way ANOVA comparing the *R^2^* coefficient values of the sham + control group, the Z-DEVD-FMK injected group with axonal imposition, and the Z-DEVD-FMK injected group without axonal imposition. There was a significant difference between groups *F*_(2,15)_ = 13.260, *p* < 0.001, with pairwise comparisons revealing that while the sham + control group and the Z-DEVD-FMK injected group without axonal imposition did not have significantly different *R^2^* coefficients, the Z-DEVD-FMK injected group with axonal targeting errors was significantly different from the sham + control group, and from the Z-DEVD-FMK injected group without targeting errors (Figure 6).

## Discussion

We have shown that cleaved caspase-3 is expressed in the developing chick auditory brainstem in a remarkable spatiotemporal pattern that follows the ascending pathway with a sequence that roughly parallels the formation of synaptic connections. At E6, when auditory VIIIth nerve axons express cleaved caspase-3, these axons have penetrated the brainstem (Molea and Rubel, 2003; Hendricks et al., 2006). At E9–10, when caspase-3 is expressed in NM axons, these axons have reached NL (Young and Rubel, 1986; Book and Morest, 1990) and have begun to form synaptic connections (Jackson et al., 1982; Hendricks et al., 2006). At E11, a period of dendritic refinement begins (Parks and Jackson, 1984), and cleaved caspase-3 expression remains strong in NL dendrites through E13. Notably, the cleaved caspase-3 expression shown here occurs before the period of programmed cell death begins. In the developing chick brainstem, a rapid decline in cell number in NM and NL begins at E11 (Rubel et al., 1976, 1981). Our TUNEL data support a lack of apoptosis prior to E11. While we did not observe many TUNEL labeled nuclei at E13, our data showed extensive cytoplasmic labeling at this age, especially in NM. This labeling pattern has been demonstrated in deafferentation-induced apoptosis in NM, and thus may be indicative of developmental cell death in these auditory nuclei (Karnes et al., 2009).

We found that in embryos treated with caspase-3 inhibitor, NL neurons failed to form a single-cell thick lamina and smaller cells intruded into the cell-free dendritic zone surrounding NL neurons. Given their size, location and ALDH1L1 immunofluorescence, these cells are likely astrocytes normally restricted to the glial margin outside NL at these ages. These morphological changes were correlated with axon targeting errors, suggesting that communication between NM axons, NL neurons and glial cells is needed for the formation of NL and its appropriate innervation.

### Nucleus Innervation and Morphology

Axonal input from NM neurons may guide NL development. NM neurons are the first to develop within the auditory brainstem (Rubel et al., 1976; Book and Morest, 1990; Cramer et al., 2000) and may influence other auditory nuclei (Hendricks et al., 2006). Prior to receiving input from the cochlea, NM neurons send projections to innervate the ventral portion of the contralateral NL. NM inputs to NL are likely specified early in development, and may guide the tonotopic identity of NL neurons and formation of hindbrain circuitry (Cramer et al., 2000; Hendricks et al., 2006).

NL dendritic morphology depends on innervation from NM. Several studies have demonstrated that when the dorsal cochlear tract (the axon tract projecting from the NM to the ventral NL) is cut, NL morphology changes (Parks and Rubel, 1975; Benes et al., 1977; Deitch and Rubel, 1984). Ventral NL dendrites become atrophied and degraded, which is reflected in decreases in dendrite density and ventral NL volume (Benes et al., 1977). Following de-innervation of the ventral NL, cells from the glial margin moved into the cell-free dendritic zone (Rubel et al., 1981). This observation suggests that axonal input is needed to maintain neuronal morphology in NL, and disruptions in the glial margin follow disruptions to the neuronal lamina. Given that axonal innervation is a significant factor in proper NL development, misrouted axons, as seen in our data, may drive malformations of NL.

### Caspase-3 Function in Axons and Dendrites

Cleaved caspase-3 influences the survival, structure and trajectory of developing axons. Apoptosis is accompanied by caspase-3 induced cleavage of cytoskeletal actin (Mashima et al., 1997, 1999). However, given that several other cytoskeletal-associated proteins are also cleaved by caspase-3, the function of caspase-3 in axons extends beyond apoptosis. Caspase-3 has been implicated in axonal growth cone guidance. In order to navigate towards their targets, axonal growth cones rely on cell surface receptors to detect chemotropic cues within their environment. Chemotropic input can then induce caspase-3 dependent degradation of cytoskeletal growth cone proteins (Campbell and Holt, 2001, 2003). In addition to directly acting on cytoskeletal proteins, caspase-3 can also cleave a host of cytoskeletal regulators, including Gap43, which is present in growth cones (Benowitz and Routtenberg, 1997; Denny, 2006). Loss of active caspase-3 may then prevent the degradation of inappropriate or misrouted axonal growth cones, resulting in errors in axonal trajectory.

Cleaved caspase-3 also influences axonal arborization. Acting in concert with caspase-9, p38, and MAPK, caspase-3 acts to localize branch points in developing retinal ganglion cell axonal arbors (Campbell and Okamoto, 2013). Caspase-3 becomes active within 5 min of branch-point formation with activity decreasing as arbors become more stable (Campbell et al., 2007; Campbell and Okamoto, 2013), and cleaves several cytoskeletal regulators to limit arbor growth (Han et al., 2013). Without activated caspase-3 present to guide where branch points may form, axons may branch in unsuitable places, and impair nucleus innervation. Despite caspase-3 blockage however, contralateral NM projecting axons did generally arrive near their NL targets, suggesting that caspase-3 influences short-range intercellular signaling rather than long-range diffusible factors.

### Axon Guidance and Nucleus Formation

The effects of caspase-3 inhibition on both NL formation and axon targeting to NL are similar to those we previously observed for Eph protein inhibition, with caspase-3 blockade showing a larger magnitude for both effects. Eph receptors and their ephrin ligands are membrane-associated molecules that operate over short distances and play an important role in the development of the auditory brainstem. Eph receptor-ephrin interactions allow for bidirectional signaling between cells (Davy et al., 1999; Lim et al., 2008; Cramer and Gabriele, 2014; Cramer and Miko, 2016), which can result in attraction to, or repulsion from a target (Cowan and Henkemeyer, 2002; Kullander and Klein, 2002). In the avian brainstem, EphA4 is expressed much more strongly in dorsal NL dendrites than in ventral dendrites. When expression of EphA4 was experimentally altered, incoming contralateral axonal inputs from the NM did not remain confined to the ventral portion of NL (Cramer et al., 2004; Huffman and Cramer, 2007), suggesting that EphA4 serves to restrict the areas along NL where NM inputs may synapse (Cramer et al., 2004). Similar mistargeting occurred when EphB2 was inhibited (Allen-Sharpley and Cramer, 2012). Interestingly, in studies of both EphA4 and EphB2 disruption, the morphological lamination of NL was also impaired (Cramer et al., 2004; Allen-Sharpley and Cramer, 2012). These observations support the hypothesis that NM axon targeting is closely related to morphogenesis in NL.

An intriguing possibility is that caspase-3 effects share signaling pathways with Eph family proteins. Eph receptors can also influence cell proliferation in a caspase-3 dependent fashion. During brain development, Eph receptor—ephrin interactions limit brain size by controlling cell proliferation. Developing telencephalon size is dependent on EphA7 binding to ephrin-A5 to initiate caspase-3 mediated cell death within the pool of progenitor cells, thus regulating brain size (Depaepe et al., 2005). Similarly, when ephrin-B3 is un-bound from its EphA4 receptor, caspase dependent cell death is initiated, during which process EphA4 itself is cleaved by a caspase-3-like caspase (Furne et al., 2009). Eph-receptors can initiate caspase-3 cleavage and there is some evidence that caspase-3 can cleave Eph receptors. Whether caspase-3 signaling interacts with Eph family proteins in axon guidance remains to be determined.

## Author Contributions

SER contributed to all of the experimental studies, data analysis and preparation of the manuscript. MRA-S initiated the project and performed the caspase-3 expression studies and analysis. KSC contributed to experimental design, to some of the anatomical studies and analysis and to manuscript preparation.

## Conflict of Interest Statement

The authors declare that the research was conducted in the absence of any commercial or financial relationships that could be construed as a potential conflict of interest.

## References

[B1] Allen-Sharpley M. R. Cramer K. S. (2012). Coordinated Eph-ephrin signaling guides migration and axon targeting in the avian auditory system. Neural Dev. 7:29. 10.1186/1749-8104-7-29 22908944 PMC3515360

[B2] Allen-Sharpley M. R. Tjia M. Cramer K. S. (2013). Selective tracing of auditory fibers in the avian embryonic vestibulocochlear nerve. J. Vis. Exp. 73:e50305. 10.3791/50305 23542875 PMC3639547

[B3] Benes F. M. Parks T. N. Rubel E. W. (1977). Rapid dendritic atrophy following deafferentation: an EM morphometric analysis. Brain Res. 122, 1–13. 10.1016/0006-8993(77)90658-8 837214

[B4] Benowitz L. I. Routtenberg A. (1997). GAP-43: an intrinsic determinant of neuronal development and plasticity. Trends Neurosci. 20, 84–91. 10.1016/s0166-2236(96)10072-2 9023877

[B5] Book K. J. Morest D. K. (1990). Migration of neuroblasts by perikaryal translocation: role of cellular elongation and axonal outgrowth in the acoustic nuclei of the chick embryo medulla. J. Comp. Neurol. 297, 55–76. 10.1002/cne.902970105 2376633

[B6] Campbell D. S. Holt C. E. (2001). Chemotropic responses of retinal growth cones mediated by rapid local protein synthesis and degradation. Neuron 32, 1013–1026. 10.1016/s0896-6273(01)00551-7 11754834

[B7] Campbell D. S. Holt C. E. (2003). Apoptotic pathway and MAPKs differentially regulate chemotropic responses of retinal growth cones. Neuron 37, 939–952. 10.1016/s0896-6273(03)00158-2 12670423

[B8] Campbell D. S. Okamoto H. (2013). Local caspase activation interacts with Slit-Robo signaling to restrict axonal arborization. J. Cell Biol. 203, 657–672. 10.1083/jcb.201303072 24385488 PMC3840933

[B9] Campbell D. S. Stringham S. A. Timm A. Xiao T. Law M. Y. Baier H.. (2007). Slit1a inhibits retinal ganglion cell arborization and synaptogenesis via Robo2-dependent and -independent pathways. Neuron 55, 231–245. 10.1016/j.neuron.2007.06.034 17640525

[B10] Carr C. E. Konishi M. (1990). A circuit for detection of interaural time differences in the brain stem of the barn owl. J. Neurosci. 10, 3227–3246. 2213141 10.1523/JNEUROSCI.10-10-03227.1990 PMC6570189

[B11] Cowan C. A. Henkemeyer M. (2002). Ephrins in reverse, park and drive. Trends Cell Biol. 12, 339–346. 10.1016/s0962-8924(02)02317-6 12185851

[B14] Cramer K. S. Bermingham-McDonogh O. Krull C. E. Rubel E. W. (2004). EphA4 signaling promotes axon segregation in the developing auditory system. Dev. Biol. 269, 26–35. 10.1016/j.ydbio.2004.01.002 15081355

[B15] Cramer K. S. Fraser S. E. Rubel E. W. (2000). Embryonic origins of auditory brain-stem nuclei in the chick hindbrain. Dev. Biol. 224, 138–151. 10.1006/dbio.2000.9779 10926755

[B12] Cramer K. S. Gabriele M. L. (2014). Axon guidance in the auditory system: multiple functions of Eph receptors. Neuroscience 277, 152–162. 10.1016/j.neuroscience.2014.06.068 25010398 PMC4164577

[B13] Cramer K. S. Miko I. J. (2016). Eph-ephrin signaling in nervous system development. F1000Res. 5:413. 10.12688/f1000research.7417.1 27092247 PMC4821289

[B16] Dalmau I. Vela J. M. González B. Finsen B. Castellano B. (2003). Dynamics of microglia in the developing rat brain. J. Comp. Neurol. 458, 144–157. 10.1002/cne.10572 12596255

[B17] Davy A. Gale N. W. Murray E. W. Klinghoffer R. A. Soriano P. Feuerstein C.. (1999). Compartmentalized signaling by GPI-anchored ephrin-A5 requires the Fyn tyrosine kinase to regulate cellular adhesion. Genes Dev. 13, 3125–3135. 10.1101/gad.13.23.3125 10601038 PMC317175

[B18] Deitch J. S. Rubel E. W. (1984). Afferent influences on brain stem auditory nuclei of the chicken: time course and specificity of dendritic atrophy following deafferentation. J. Comp. Neurol. 229, 66–79. 10.1002/cne.902290106 6490976

[B19] Denny J. B. (2006). Molecular mechanisms, biological actions and neuropharmacology of the growth-associated protein GAP-43. Curr. Neuropharmacol. 4, 293–304. 10.2174/157015906778520782 18654638 PMC2475799

[B20] Depaepe V. Suarez-Gonzalez N. Dufour A. Passante L. Gorski J. A. Jones K. R.. (2005). Ephrin signalling controls brain size by regulating apoptosis of neural progenitors. Nature 435, 1244–1250. 10.1038/nature03651 15902206

[B21] Ertürk A. Wang Y. Sheng M. (2014). Local pruning of dendrites and spines by caspase-3-dependent and proteasome-limited mechanisms. J. Neurosci. 34, 1672–1688. 10.1523/jneurosci.3121-13.2014 24478350 PMC6827581

[B22] Fan W. Dai Y. Xu H. Zhu X. Cai P. Wang L.. (2014). Caspase-3 modulates regenerative response after stroke. Stem Cells 32, 473–486. 10.1002/stem.1503 23939807

[B23] Furne C. Ricard J. Cabrera J. R. Pays L. Bethea J. R. Mehlen P.. (2009). EphrinB3 is an anti-apoptotic ligand that inhibits the dependence receptor functions of EphA4 receptors during adult neurogenesis. Biochim. Biophys. Acta 1793, 231–238. 10.1016/j.bbamcr.2008.09.009 18948148 PMC2631096

[B24] Han M. H. Jiao S. Jia J. M. Chen Y. Chen C. Y. Gucek M.. (2013). The novel caspase-3 substrate Gap43 is involved in AMPA receptor endocytosis and long-term depression. Mol. Cell. Proteomics 12, 3719–3731. 10.1074/mcp.m113.030676 24023391 PMC3861719

[B25] Hendricks S. J. Rubel E. W. Nishi R. (2006). Formation of the avian nucleus magnocellularis from the auditory anlage. J. Comp. Neurol. 498, 433–442. 10.1002/cne.21031 16874806

[B26] Huffman K. J. Cramer K. S. (2007). EphA4 misexpression alters tonotopic projections in the auditory brainstem. Dev. Neurobiol. 67, 1655–1668. 10.1002/dneu.20535 17577206

[B27] Ishizaki Y. Jacobson M. D. Raff M. C. (1998). A role for caspases in lens fiber differentiation. J. Cell Biol. 140, 153–158. 10.1083/jcb.140.1.153 9425163 PMC2132591

[B28] Jackson H. Hackett J. T. Rubel E. W. (1982). Organization and development of brain stem auditory nuclei in the chick: ontogeny of postsynaptic responses. J. Comp. Neurol. 210, 80–86. 10.1002/cne.902100109 7130472

[B29] Jacobson M. D. (1997). Programmed cell death: a missing link is found. Trends Cell Biol. 7, 467–469. 10.1016/s0962-8924(97)01182-3 17709009

[B30] Karnes H. E. Kaiser C. L. Durham D. (2009). Deafferentation-induced caspase-3 activation and DNA fragmentation in chick cochlear nucleus neurons. Neuroscience 159, 804–818. 10.1016/j.neuroscience.2008.12.031 19166907

[B31] Kuida K. Zheng T. S. Na S. Kuan C. Yang D. Karasuyama H.. (1996). Decreased apoptosis in the brain and premature lethality in CPP32-deficient mice. Nature 384, 368–372. 10.1038/384368a0 8934524

[B32] Kullander K. Klein R. (2002). Mechanisms and functions of Eph and ephrin signalling. Nat. Rev. Mol. Cell Biol. 3, 475–486. 10.1038/nrm856 12094214

[B33] Lim B. K. Matsuda N. Poo M. M. (2008). Ephrin-B reverse signaling promotes structural and functional synaptic maturation *in vivo*. Nat. Neurosci. 11, 160–169. 10.1038/nn2033 18193042

[B35] Mashima T. Naito M. Noguchi K. Miller D. K. Nicholson D. W. Tsuruo T. (1997). Actin cleavage by CPP-32/apopain during the development of apoptosis. Oncogene 14, 1007–1012. 10.1038/sj.onc.1200919 9070648

[B34] Mashima T. Naito M. Tsuruo T. (1999). Caspase-mediated cleavage of cytoskeletal actin plays a positive role in the process of morphological apoptosis. Oncogene 18, 2423–2430. 10.1038/sj.onc.1202558 10229193

[B36] Mayordomo R. Valenciano A. I. de la Rosa E. J. Hallböök F. (2003). Generation of retinal ganglion cells is modulated by caspase-dependent programmed cell death. Eur. J. Neurosci. 18, 1744–1750. 10.1046/j.1460-9568.2003.02891.x 14622209

[B37] Molea D. Rubel E. W. (2003). Timing and topography of nucleus magnocellularis innervation by the cochlear ganglion. J. Comp. Neurol. 466, 577–591. 10.1002/cne.10896 14566951

[B38] Morishita H. Makishima T. Kaneko C. Lee Y. S. Segil N. Takahashi K.. (2001). Deafness due to degeneration of cochlear neurons in caspase-3-deficient mice. Biochem. Biophys. Res. Commun. 284, 142–149. 10.1006/bbrc.2001.4939 11374883

[B39] Oomman S. Strahlendorf H. Finckbone V. Strahlendorf J. (2005). Non-lethal active caspase-3 expression in Bergmann glia of postnatal rat cerebellum. Dev. Brain Res. 160, 130–145. 10.1016/j.devbrainres.2005.07.010 16226814

[B40] Overholt E. M. Rubel E. W. Hyson R. L. (1992). A circuit for coding interaural time differences in the chick brainstem. J. Neurosci. 12, 1698–1708. 1578264 10.1523/JNEUROSCI.12-05-01698.1992 PMC6575867

[B41] Parks T. N. Jackson H. (1984). A developmental gradient of dendritic loss in the avian cochlear nucleus occurring independently of primary afferents. J. Comp. Neurol. 227, 459–466. 10.1002/cne.902270315 6480902

[B42] Parks T. N. Rubel E. W. (1975). Organization and development of brain stem auditory nuclei of the chicken: organization of projections from N. magnocellularis to n. laminaris. J. Comp. Neurol. 164, 435–448. 10.1002/cne.901640404 1206128

[B43] Person A. L. Cerretti D. P. Pasquale E. B. Rubel E. W. Cramer K. S. (2004). Tonotopic gradients of Eph family proteins in the chick nucleus laminaris during synaptogenesis. J. Neurobiol. 60, 28–39. 10.1002/neu.10330 15188270

[B44] Pompeiano M. Blaschke A. J. Flavell R. A. Srinivasan A. Chun J. (2000). Decreased apoptosis in proliferative and postmitotic regions of the caspase 3-deficient embryonic central nervous system. J. Comp. Neurol. 423, 1–12. 10.1002/1096-9861(20000717)423:1<1::aid-cne1>3.0.co;2-s 10861532

[B45] Roth K. A. Kuan C. Haydar T. F. D’Sa-Eipper C. Shindler K. S. Zheng T. S.. (2000). Epistatic and independent functions of caspase-3 and Bcl-X_L_ in developmental programmed cell death. Proc. Natl. Acad. Sci. U S A 97, 466–471. 10.1073/pnas.97.1.466 10618441 PMC26686

[B46] Rubel E. W. Smith D. J. Miller L. C. (1976). Organization and development of brain stem auditory nuclei of the chicken: ontogeny of N. magnocellularis and N. laminaris. J. Comp. Neurol. 166, 469–489. 10.1002/cne.901660408 1270618

[B47] Rubel E. W. Smith Z. D. Steward O. (1981). Sprouting in the avian brainstem auditory pathway: dependence on dendritic integrity. J. Comp. Neurol. 202, 397–414. 10.1002/cne.902020309 7298906

[B48] Salvesen G. S. Dixit V. M. (1997). Caspases: intracellular signaling by proteolysis. Cell 91, 443–446. 10.1016/s0092-8674(00)80430-4 9390553

[B49] Smith D. J. Rubel E. W. (1979). Organization and development of brain stem auditory nuclei of the chicken: dendritic gradients in nucleus laminaris. J. Comp. Neurol. 186, 213–239. 10.1002/cne.901860207 447882

[B50] Tabor K. M. Wong R. O. Rubel E. W. (2011). Topography and morphology of the inhibitory projection from superior olivary nucleus to nucleus laminaris in chickens (Gallus gallus). J. Comp. Neurol. 519, 358–375. 10.1002/cne.22523 21165979 PMC3299086

[B51] Takahashi K. Kamiya K. Urase K. Suga M. Takizawa T. Mori H.. (2001). Caspase-3-deficiency induces hyperplasia of supporting cells and degeneration of sensory cells resulting in the hearing loss. Brain Res. 894, 359–367. 10.1016/s0006-8993(01)02123-0 11251216

[B52] Wang J. Lenardo M. J. (2000). Roles of caspases in apoptosis, development, and cytokine maturation revealed by homozygous gene deficiencies. J. Cell Sci. 113, 753–757. 10671365 10.1242/jcs.113.5.753

[B53] Wang Y. Rubel E. W. (2008). Rapid regulation of microtubule-associated protein 2 in dendrites of nucleus laminaris of the chick following deprivation of afferent activity. Neuroscience 154, 381–389. 10.1016/j.neuroscience.2008.02.032 18440716 PMC2693030

[B54] Young S. R. Rubel E. W. (1986). Embryogenesis of arborization pattern and topography of individual axons in N. laminaris of the chicken brain stem. J. Comp. Neurol. 254, 425–459. 10.1002/cne.902540402 3805357

[B55] Zhang Y. Goodyer C. LeBlanc A. (2000). Selective and protracted apoptosis in human primary neurons microinjected with active caspase-3, -6, -7 and -8. J. Neurosci. 20, 8384–8389. 11069945 10.1523/JNEUROSCI.20-22-08384.2000 PMC6773170

[B56] Zou H. Henzel W. J. Liu X. Lutschg A. Wang X. (1997). Apaf-1, a human protein homologous to *C. elegans* CED-4, participates in cytochrome c-dependent activation of caspase-3. Cell 90, 405–413. 10.1016/s0092-8674(00)80501-2 9267021

